# Green Tea Extract (GTE) improves differentiation in human osteoblasts during oxidative stress

**DOI:** 10.1186/1476-9255-11-15

**Published:** 2014-05-18

**Authors:** Helen Vester, Nina Holzer, Markus Neumaier, Schyschka Lilianna, Andreas K Nüssler, Claudine Seeliger

**Affiliations:** 1 Department of Trauma Surgery, Technical University Munich, MRI, Munich, Germany; 2 Department of experimental Trauma Surgery, Technical University Munich, MRI, Munich, Germany; 3 Department of Trauma Surgery, Eberhard Karls University Tubingen, Tubingen, Germany

**Keywords:** Green Tea Extract, Primary human osteoblasts, Oxidative stress, Differentiation

## Abstract

**Background:**

Oxidative stress is involved in the pathogenesis of bone diseases such as osteoporosis, which has a high coincidence with fractures in elderly. Several studies showed positive effects of herbal bioactive substances on oxidative stress. This study analyses the effect of green tea extract (GTE) Sunphenon 90LB on primary human osteoblasts differentiation and viability during H_2_O_2_-induced oxidative stress. Moreover, it was analyzed, whether GTE acts during the HO-1 signaling pathway.

**Methods:**

Human osteoblasts were isolated from femoral heads of patients undergoing total hip replacement. Beneficial effects of GTE on osteoblasts were examined in a dose- and time-dependent manner. Furthermore, GTE was given before, simultaneous with and after induction of oxidative stress with 1 mM H_2_O_2_ to simulate prophylactic, acute and therapeutic use, respectively. Cell damage was measured by LDH leakage and cell viability by MTT assay. Flow cytometry was applied to measure formation of Reactive Oxygen Species by using 2`7`-dichlorofluorescein-diacetate. The formation of Extracellular Matrix after differentiation with GTE supplementation during oxidative stress was visualized with von Kossa and Alizarin Red staining. Last one was additionally photometrically quantified. To assess the effects of H_2_O_2_ and GTE on the osteogenic genes, RT-PCR was performed. To evaluate the intramolecular influence of GTE after the stimulation the protein levels of HO-1 were analyzed.

**Results:**

Stimulation of primary human osteoblasts with low doses of GTE during oxidative stress over 21 days improved mineralization. Furthermore, GTE supplementation in combination with H_2_O_2_ leads to a higher gene expression of osteocalcin and collagen1α1 during osteoblasts differentiation. Both are important for bone quality. Pre-incubation, co-incubation and post-incubation of osteoblasts with high doses of GTE protect the osteoblasts against acute oxidative stress as shown by increased cell viability, decreased LDH leakage, and reduced production of intracellular free radicals. Functional analysis revealed an increased HO-1 protein synthesis after stimulation with GTE.

**Conclusions:**

Incubation of human primary osteoblasts with GTE significantly reduces oxidative stress and improves cell viability. GTE also has a beneficial effect on ECM production which might improve the bone quality. Our findings suggest that dietary supplementation of GTE might reduce inflammatory events in bone-associated diseases such as osteoporosis.

## Background

Bone is a tissue subjected to continuous rebuilding processes. Imbalances between new bone formation caused by osteoblasts and bone resorption triggered by osteoclasts result in impaired bone quality or even osteopenia/osteoporosis, which renders the individual highly susceptible to fractures [1,2]. Poor bone quality and osteopenia (also called low bone mass) have been reported in patients with chronic inflammatory diseases, such as rheumatoid arthritis or inflammatory bowel disease [3]. Chronic systemic inflammation-induced bone loss has been associated with high levels of oxidative stress in animals. Shen et al. showed that lipopolysaccharide administration in rats leads to a decrease in femur mineral content and density. Thus, oxidative stress seems to be related to bone loss [4,5].

Reactive Oxygen Species (ROS), e.g. hydrogen peroxides or superoxides can induce several molecular alterations in cellular components, leading to changes in cell morphology, viability and function. This is due to lesions of DNA strands, protein cross-links and side-chain oxidation. Oxidative stress results from an excess of ROS-disturbing physiological cell cycles or from environmental stimuli perturbing the normal cellular redox system, thereby shifting cells into a state of oxidative stress [6].

Former studies could show that oxidative stress decreases the quantity and quality of osteoblasts [7,8] and increases the apoptosis of osteoblasts and osteocytes [9]. Additionally in the case of osteoclasts, oxidative stress increases their differentiation and function, which leads to reduced bone mass formation [10,11].

Augmentation of endogenous oxidative defence seems to be one possibility to prevent the organism from ROS-mediated cellular injury. Besides increased dietary intake of antioxidants, such as vitamins A, C and E, attention has been paid recently to non-vitamin antioxidants, such as phenolic compounds, which also might support cellular defence mechanisms. Red wine polyphenols or soya phytoestrogens are very well-known antioxidants [12-14]. Tea also contains various supplements including antioxidants and was famous for its anti-inflammatory and antioxidative properties even in ancient times. Especially green tea and its polyphenolic compounds - catechins - are known to prevent oxidative stress [15-17]. The major green tea catechins are epicatechin (EC), epigallocatechin (EGC), epicatechin gallate (ECG), and epigallocatechin gallate (EGCG) [18-20]. In our study we used the innovated decaffeinated Green Tea Extract Sunphenon LG90 (GTE) containing more than 80% polyphenols, thereof more than 80% catechins, more than 40% epigallocatechin and less than 1% caffeine. It was already reported to have positive effects *in vivo* on warm ischemia/reperfusion (I/R) injury in rat livers [21]. Preconditioning with GTE ameliorates I/R injury, decreases lactate dehydrogenase (LDH) release and hepatic necrosis. Moreover, GTE inhibits the production of proinflammatory cytokines such as TNF-α or IL-1 in this model. Former *in vitro* studies performed by our team with human osteoblasts treated with cigarette smoke medium showed an improvement of cell viability after GTE application, which can be linked to elevated heme-oxygenase expression [22]. Moreover, underlying intracellular mechanisms for the antioxidative effect of GTE are still unclear. There is increasing evidence that heme oxygenase-1 (HO-1) induction represents an adaptive response or enhanced resistance against various oxidative stresses. The transcription factor nuclear factor erythroid 2-related factor 2 (Nrf2) is a critical regulator of HO-1, achieved by binding to the antioxidant response element (ARE). Activation of Nrf2 by phosphorylation leads to synthesis of several antioxidative mediators. Hyon et al. could show, that Nrf2 deprivation leads to an increase of oxidative stress and osteoclast differentiation by RANKL activation [23,24]. In this context polyphenols have been reported to up-regulate HO-1 expression by activation Nrf2 to bind the antioxidant response element in the HO-1 gene promoter region [25]. As one major pathway for protecting the cell against oxidative stress we decided to analyse whether this pathway is influenced by GTE or could explain its protective effect.

So far, some studies could show beneficial effects of different GTE on osteoblasts, mostly isolated from rats or mice [25,26] and GTE seem to be a promising dietary supplement for preventing bone loss [27]. For clinical application, however, it is of great interest to know whether GTE also has beneficial effects on human primary osteoblasts. Moreover, referring to bone quality, it is necessary to analyse the mineralization, which is responsible for bone stability. Therefore, the aim of this study was to investigate the influence of GTE on oxidative stress in bone cells and to analyse potential underlying signalling pathways.

## Methods

GTE Sunphenon 90LB was obtained from Taiyo International (Fiderstadt, Germany). Fetal calf serum (FCS gold), penicillin, streptomycin and phosphate buffered saline (PBS) were purchased from PAA Laboratories GmbH (Pasching, Austria). Collagenase type II was obtained from Biochrom (Berlin, Germany). Cell culture medium and all other chemicals were purchased from Sigma (Munich, Germany).

### Isolation and culture of primary human osteoblasts

Primary human osteoblasts were isolated from femur heads of patients undergoing total hip replacement, with their informed consent. This study was approved by the local ethical review committee of the Faculty of Medicine of the Technical University of Munich (project number 2033/08). The study was performed according to the declaration of Helsinki in its newest version. Briefly, cancellous bone was removed mechanically from the femur head, washed 5 times with PBS and digested for 1 h at 37°C with an equal volume of 0.07% Collagenase II in PBS. The enzymatic reaction was stopped by osteoblast culture medium (MEM/Ham's F12 with l-glutamine, 10% FCS, 100 U/ml penicillin, 100 μg/ml streptomycin, 50 μM L-ascorbate-2-phosphate and 50 μM β-glycerol-phosphate). Bone pieces were transferred to a cell culture flask with 25 ml cell culture medium. The supernatant was centrifuged at 650 × g for 10 minutes. Afterwards, the supernatant was aspirated; the cell pellets were resuspended and distributed to flasks. Medium was changed every 4-5 days. After two weeks the osteoblasts were growing out of the bone pieces [28]. The cells were expanded and used for experiments from passage 3 onwards at a density of 2.0 × 10^4^ cells/cm^2^.

### MTT viability assay, LDH assay

For MTT assay, cell culture medium was replaced with 0.5 mg/ml MTT solution per well. Osteoblasts were incubated for the next 1.5 h at 37°C, 5% CO_2_, allowing viable cells to metabolize the yellow MTT to dark purple formazan crystals, which were dissolved with an equal volume of MTT solubilisation solution (10% SDS, 0.6% acetic acid in DMSO). Absorbance was measured at 570 nm and 690 nm as a reference using a FLUOstar Omega fluorometer, BMG Labtech (Offenburg, Germany).

To evaluate cellular damage, the content of lactate dehydrogenase (LDH) in the culture supernatants was measured using a commercially available reaction kit (Analyticon Biotechnologies, Lichtenfels, Germany).

### ROS formation measurement

For ROS measurement all cells were detached by trypsinization and incubated with 10 μM 2', 7'-dichlorfluorescein-diacetate (DCFH-DA) in serum-free culture medium for 30 min at 37°C and 5% CO_2_. ROS measurement is designed to detect the reactive oxygen species production in various cell lines. During oxidative stress the added chemical compound DCFH-DA will be catalysed to 2`7`- dichlorofluorescein (DCFH) and this can be detected by flow cytometric analysis at ex/em = 488/527 nm. The cell pellet was stimulated with GTE in the pre-incubation setting for 1 h, trypsinized and washed 3 times with PBS. The cells were treated with 1 mM H_2_O_2_ for the next 15 min [29]. For the post-incubation setting, the cells were first treated with H_2_O_2_, subsequently washed and stimulated with GTE for 1 h. The ROS measurement was not applicable for co-incubation setting due to interaction between GTE and H_2_O_2_. The acquisition of the fluorescence signal was performed directly after treatment in the FITC channel on FACS Canto II (BD Biosciences, San Jose, USA). Flow Jo (Treestar Inc., Ashland, USA) was used for the calculation of the produced DCFH of the cells.

### Osteogenic differentiation

For osteogenic differentiation, the cells were cultured for 21 days in differentiation medium (DMEM low glucose, 5% FCS, 2 mM L-glutamine, 100 U/ml penicillin, 100 μg/ml streptomycin, 100 μM L-ascorbate-2-phosphate, 10 mM β-glycerol-phosphate, 25 mM HEPES, 1.5 mM CaCl_2_, 100 nM dexamethasone). During this period, the cells were stimulated six times with/without 50 μM H_2_O_2_ and 0.01 μg/ml, 0.1 μg/ml and 1 μg/ml of GTE.

### Alizarin red staining

After the osteogenic differentiation, cells were washed with PBS and fixed for 15 min with ice-cold methanol. Osteoblasts were stained with 0.5% alizarin red solution (pH = 4) for 10 min at RT and subsequently washed 3 times with tab water. Pictures were taken with HP scanner, staining precipitates were dissolved with 10% cetylpyridinium chloride solution and the optical densities of samples and standard curve were measured in the fluorometer at 562 nm [30].

### Van Kossa staining

For the staining, cells were washed with PBS and fixed for 1 h with ice cold ethanol. Afterwards excessive ethanol was washed out 3 times with tap water and the cells were stained with 3% silver-nitrate for 30 min at RT. Subsequently cells were washed 3 times with tap water and covered with 1% pyragallolsolution and afterwards with 5% sodiumthiosulfate solution for fixation. The nuclei were stained with kernechtred. Pictures were taken with HP scanner.

### Real-time PCR

Total RNA of differentiated osteoblasts, with or without exposure to H_2_O_2_ and GTE, was extracted using Trizol reagent, according to the manufacturer’s recommendations (PeqLab, Erlangen, Germany). The amount and purity of RNA was determined by photometry. RNA integrity was examined by agarose gel electrophoresis. RNA was transcribed to first-strand cDNA using the First Strand cDNA Synthesis Kit (Fermentas, St. Leon-Rot, Germany). The sequence of both the forward and reverse primers and conditions for RT-PCR are listed in Table 1. To assess the effects of H_2_O_2_ and GTE on the genes, PCR amplification was performed using SYBR Green real-time PCR master mix with a CFX 96 Touch Real-Time PCR System (Bio-Rad, München, Germany). Expression level of each gene was determined as the cycle number by real-time PCR, with their levels normalized to that of the housekeeping gene Tubulin-β (TUB-B) using the ΔΔCT method.

**Table 1:** Primer sequences and PCR conditions, OC = osteocalcin, COL = collagen, TUB-B = tubulin β

		**Sequence 5´ to 3**	
	**Accession no.**	**Forward primer**	**Reverse primer**
OC	NM_199173.3	CCAGCGGTGCAGAGTCCAGC	GACACCCTAGACCGGGCCGT
COL1A1	NM_000088.3	AGCGGACGCTAACCCCCTCC	CAGACGGGACAGCACTCGCC
TUB-B	NM_001069.2	GAGGGCGAGGACGAGGCTTA	TCTAACAGAGGCAAAACTGAGCACC

### Western blot analysis

After stimulation of the cells with 200 μg/ml GTE, 1 mM H_2_O_2_ and as described before with 25 μM zinc protoporphyrine (ZNPP9) total protein was collected and measured after standard protocol [31]. Briefly, the cells were lysed in ice cold RIPA lysis buffer (50 mM TRIS; 250 mM NaCl; 2% Nonidet-P40; 2.5 mM EDTA; 0.1% SDS; 0.5% DOC; complete protease inhibitor; 1% phosphatase inhibitor, Na3VO4 (100 mM), PMSF (50 mM), pH = 7.2). Protein concentration was determined by the method of Lowry [32]. 40 μg total protein was separated by 10% SDS PAGE and transferred to nitrocellulose membranes (Roth, Karlsruhe, Germany). Antibody sources are summarized in Table 2. Membranes were incubated with the first antibody over night at 4°C in the dark. Next day after washing the incubation with second antibody for 1 hour at room temperature followed. The development of the membrane was realized via chemiluminescence reaction. For the detection and densitometric analysis of the signals the ChemiDoc MP imaging system were used (Bio-Rad, Munich, Germany).

**Table 2:** Protein description and using conditions

**Name**	**Host species**	**Size (kDa)**	**Dilution**	**Company**
Β actin	Mouse	43 kDa	1:1000	Merck Milipore
HO-1	Rabbit	28 kDa	1:1000	Cell Signalling

### Statistics

Results are expressed as mean ± SEM of at least 3 independent experiments (N ≥ 3) measured as triplicates (n = 3). One way analysis of variance (ANOVA) with Bonferroni’s multiple comparison test was performed using the GraphPad Prism software (GraphPad Software, San Diego, CA). p < 0.05 was considered statistically significant.

## Results

### Effect of GTE on osteoblast culture

**Figure 1:**
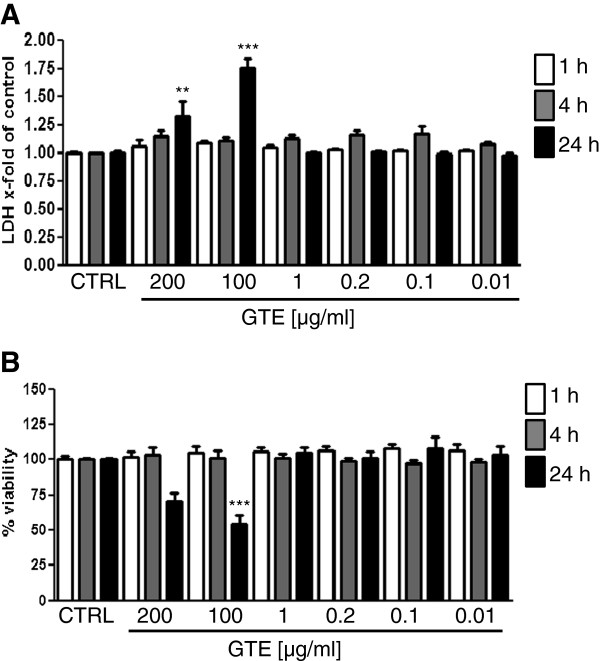
**Effect of different GTE concentrations on the viability of primary human osteoblasts.** Osteoblasts were stimulated with GTE for 1, 4 and 24 h, respectively. LDH release **(A)** and viability via MTT assay **(B)** were measured afterwards. Bars represent mean ± s.e.m., N = 3, n = 3. **p < 0.01, ***p < 0.001. (ANOVA/Bonferroni).

To investigate the effects of GTE, we tested different doses of extract on primary human osteoblasts. As shown in Figure 1A, GTE has a toxic effect on cells as assessed by LDH leakage. Only the use of 100 μg/ml and 200 μg/ml GTE for 24 h leads to a significant increase of LDH up to 1.75- and 1.33-fold in comparison to untreated cells, respectively. However, no increase of LDH release could be detected during 1 h and 4 h of stimulation in all GTE concentrations as compared to control cells. Comparable effects were obtained with the MTT cytotoxicity assay, with which the metabolic activity of the cells was measured. After 24 h of stimulation, the highest GTE concentrations 100 μg/ml and 200 μg/ml result in a decrease of cell viability of up to 45.48 ± 6.28% and 30.14 ± 6.61%, respectively (Figure 1B). All tested GTE concentrations show no detectable effects on the cell viability during 1 h and 4 h of stimulation time. In the long-time experiments, which take up to 21 days, the GTE concentrations from 100 μg/ml and 200 μg/ml show toxic effects on osteoblast cultures (data not shown). Therefore, we decided to use 100 μg/ml and 200 μg/ml GTE for the short-term experiments (up to 4 h) and 0.01 μg/ml, 0.1 μg/ml and 1 μg/ml of GTE for long-term incubation (up to 21 days).

### GTE pre-incubation significantly reduces oxidative stress

**Figure 2:**
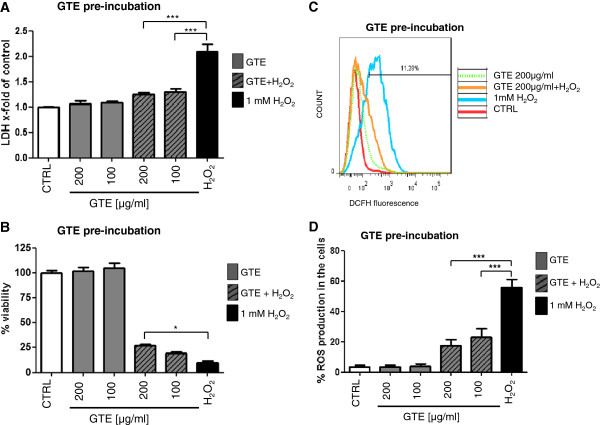
**GTE pre-incubation protects primary human osteoblasts against oxidative stress.** Osteoblasts were pre- incubated with GTE for 4 h, afterwards oxidative stress was induced with 1 mM H_2_O_2_ for the next 1 h. LDH release **(A)** and viability **(B)** were measured photometrically (N = 3, n = 3). ROS production was measured via flow cytometry, whereby the 11.9% present the DCFH positive cells **(C, D)**. Osteoblasts were pre-incubated with GTE for 1 h and subsequently for 15 min. with 1 mM H_2_O_2_ (N = 3, n = 2). Bars represent mean ± s.e.m. of three independent experiments. *p < 0.05, ***p < 0.001 (ANOVA/Bonferroni), DCFH (2`7`- dichlorofluorescein).

Human primary osteoblasts were pre-incubated with 100 μg/ml and 200 μg/ml GTE for 4 h. Afterwards, the medium was changed and the cells were treated for 1 h with 1 mM H_2_O_2_. This concentration of H_2_O_2_ was found to induce a significant level of oxidative stress in the cells (data not shown). As shown in Figure 2A, H_2_O_2_ treatment increased the LDH release up to 2.08 ± 0.15 times compared to untreated cells. The pre-treatment with 100 μg/ml and 200 μg/ml GTE protected osteoblasts from this toxic effect and reduced LDH release significantly down by 1.29 ± 0.06 and 1.25 ± 0.04 times, respectively. Similar results could be obtained with the MTT viability assay (Figure 2B). The amount of vivid cells was significantly decreased after incubation with H_2_O_2_ by up to 10.1 ± 1.46%. Nevertheless, pre-incubation with 200 μg/ml GTE caused a significant increase of cell viability of up to 26.51 ± 1.68%. No protective effects against oxidative stress caused by H_2_O_2_ treatment could be detected with concentrations below 100 μg/ml GTE.These results were supported by the analysis of ROS production in flow cytometry (Figure 2C and 2D). We could show a significant decrease of free radicals due to pre-incubation of the osteoblasts with 100 and 200 μg/ml of green tea extract (from 55.82 ± 5.24% to 23.16 ± 5.51% and 17.65 ± 3.82%, respectively). GTE concentrations lower than 100 μg/ml showed no protective abilities.

### Co-incubation with GTE protects cells against oxidative stress

**Figure 3:**
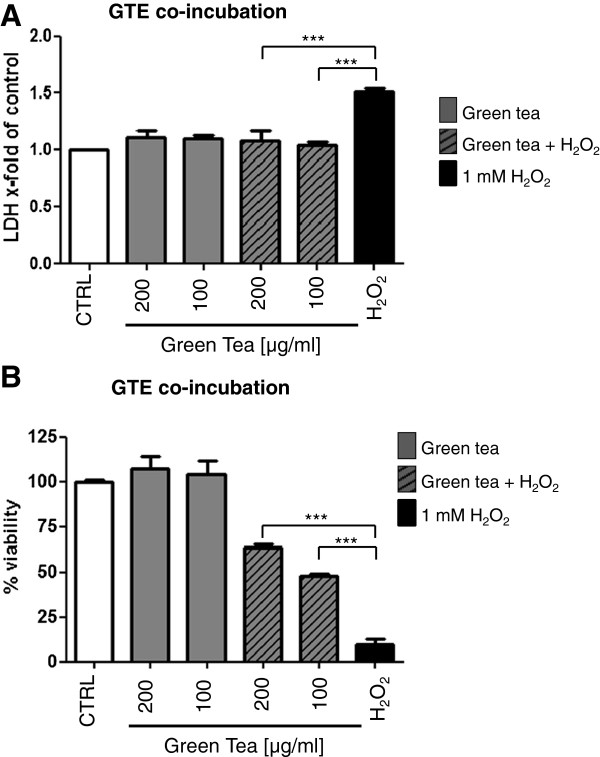
**Co-incubation with GTE can improve the viability of primary osteoblasts during oxidative stress.** Osteoblasts were simultaneously treated with GTE and 1 mM H_2_O_2_ for 4 h, afterwards LDH release **(A)** and a MTT assay **(B)** were performed (N = 3, n = 3). Bars represent mean ± s.e.m. **p < 0.01, ***p < 0.001 (ANOVA/Bonferroni).

GTE shows the same positive effects on LDH release during 4 h of co-incubation with H_2_O_2_ as pre-incubation. Figure 3A shows the significant decrease of LDH release after co-incubation with H_2_O_2_ and 100 or 200 μg/ml of GTE from 1.51 ± 0.03 to 1.03 ± 0.03 and 1.08 ± 0.09 times, respectively. This coincides with a significant increase of osteoblast viability after co-incubation with H_2_O_2_ and 100 or 200 μg/ml of GTE from 10.09 ± 2.75% up to 47.74 ± 1.67 and 63.67 ± 2.02%, respectively (Figure 3B).

### Post-incubation with GTE protects cells against oxidative stress

**Figure 4:**
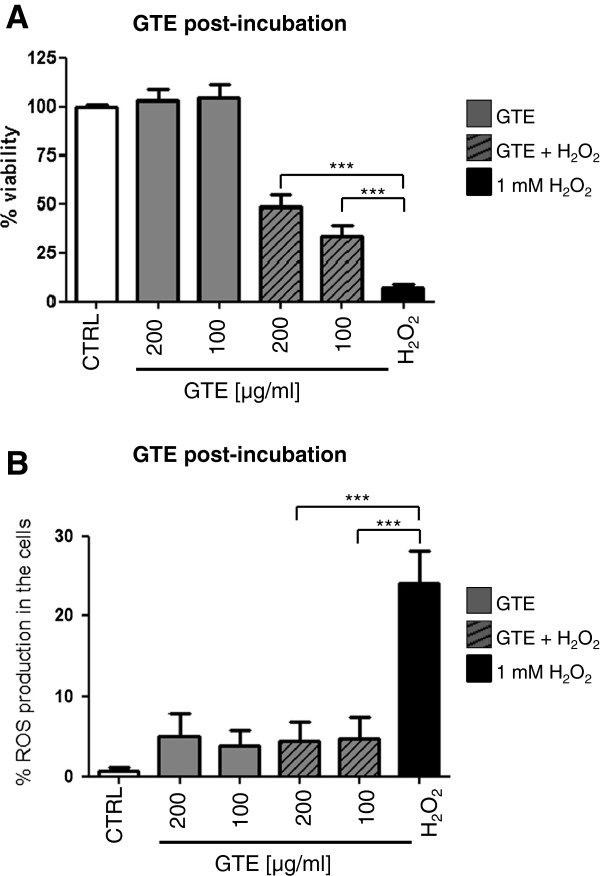
**Post-incubation with GTE recovers osteoblast viability and reduces ROS production after induction of oxidative stress.** Osteoblasts were treated for 1 h with 1 mM H_2_O_2_ followed by the stimulation with GTE for 4 h. Cell viability was measured via MTT assay **(A)**. For flow cytometry, the osteoblasts were treated for 15 min. with H_2_O_2_ and stimulated with GTE for the next hour **(B)** (N = 3, n = 2). Bars represent mean ± s.e.m. ***p < 0.001 (ANOVA/Bonferroni).

In this setup, as expected, the osteoblasts were severely damaged by oxidative stress (Figure 4A). Interestingly, post-incubation with 100 and 200 μg/ml GTE for the next 4 h resulted in a significant increase of cell viability analysed via MTT assay from 7.08 ± 2.11% up to 33.24 ± 5.65 and 48.27 ± 6.66%, respectively. A significant decrease of ROS formation could be detected after post-incubation with 100 and 200 μg/ml of GTE in the flow cytometry measurement (Figure 4B). With GTE concentrations lower than 100 μg/ml, no rescue effects were detectable (data not shown).

### GTE stimulates formation of mineralized matrix in primary human osteoblasts and increases the expression of pro-osteogenic genes during oxidative stress

**Figure 5:**
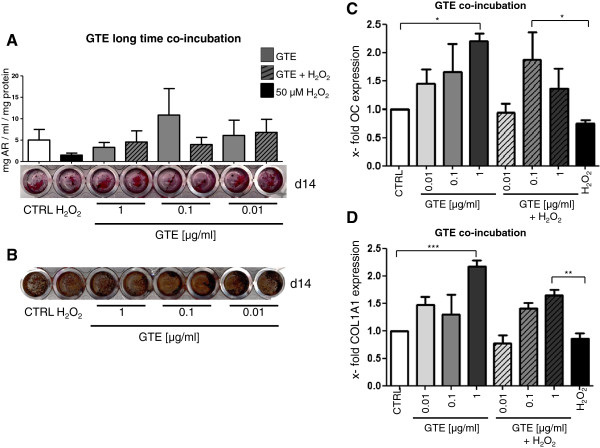
**Long-time co-incubation of primary osteoblasts with GTE improves osteogenic differentiation and expression of osteogenic genes.** Osteoblasts were stimulated for 14 **(A)** with 1 μg/ml, 0.1 μg/ml and 0.01 μg/ml GTE combined with 50 μM H_2_O_2_. Mineralization was visualized with alizarin red and afterwards quantified. Also a von Kossa staining was prepared to visualize the mineralization **(B)** (N = 4, n = 2). After 7 days stimulation with GTE combined with 50 μM H_2_O_2_, gene expression of osteocalcin **(C)** and collagen1A1 **(D)** were measured via qPCR. (N = 3, n = 3). Bars represent mean ± s.e.m, *p < 0.05, **p < 0.01, ***p < 0.001 (ANOVA/Bonferroni).

To induce oxidative stress in long-time experiments we used 50 μM H_2_O_2_. This concentration of H_2_O_2_ was found to reduce the viability of osteoblasts up to 30% over 21 days of stimulation (data not shown). According to the toxicity tests (Figure 1A, B) we also reduced the GTE concentration for up to 21 days stimulation to 0.01, 0.1 and 1 μg/ml. After 7 days no changes in development of mineralized matrix, neither in untreated cells, nor in cells simultaneously stimulated with GTE and H_2_O_2_ (data not shown) could be detected. Alizarin red staining and quantification confirmed the formation of mineralized matrix after 14 days differentiation. H_2_O_2_ alone reduced the matrix development from 5.02 ± 2.46 in untreated cells to 1.51 ± 0.46 mg/ml alizarin red/mg protein, whereas 0.01 and 0.1 μg/ml GTE increase the alizarin content up to 6.08 ± 3.57 and 10.85 ± 6.17 mg/ml alizarin red/mg protein, respectively (Figure 5A). Moreover 10 and 100 ng/ml GTE were able to counteract the negative effects of H_2_O_2_ and enhance matrix formation up to 6.81 ± 3.05 and 3.98 ± 1.66 mg/ml alizarin red/mg protein. 1 μg/ml GTE alone and in combination with H_2_O_2_ has no positive effects on mineralized matrix formation. The positive effects of GTE on mineralized matrix formation were seen by van Kossa staining as shown in Figure 5B. GTE in low concentration in this setting improved the mineralized matrix formation, whereas H_2_O_2_ alone reduced it. Moreover, 1 μg/ml, 0.1 μg/ml and 0.01 μg/ml GTE enhanced the mineralized matrix development despite H_2_O_2_ treatment.

Human osteoblasts expressed bone-related genes such as osteocalcin and collagen1A1 to a higher extent after stimulation with GTE after 7 days treatment (Figure 5C, 5D). The significant beneficial effect of GTE alone was detected after the application of 0.01, 0.1 and 1 μg/ml on both expression of osteocalcin and collagen1A1. H_2_O_2_ alone reduced the expression of both genes; this negative effect can be completely reversed by co-incubation with GTE. 0.1 μg/ml and 1 μg/ml GTE were able to increase the expression of osteocalcin up to 83.30% and 38.50% and the expression of collagen1A1 up to 41.20% and 64.3% during exposition of the osteoblasts to oxidative stress.

### GTE increased HO-1 protein synthesis

**Figure 6:**
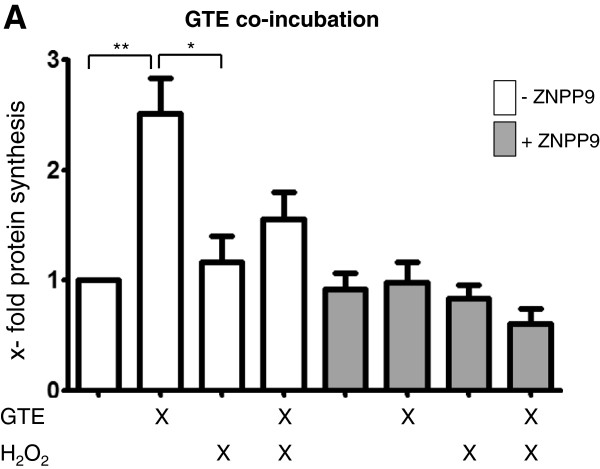
**Functional analysis of GTE on the HO-1 pathway.** Osteoblasts were stimulated for 0.5 h with 200 μg/ml GTE combined with 1 μM H_2_O_2_ and/or 25 μM ZNPP9. As controls served unstimulated cells (N = 3, n = 1). Bars represent mean ± s.e.m, *p < 0.05, (ANOVA/Bonferroni).

To proof whether the protective effect of GTE is dependent on HO-1 expression, we stimulate osteoblasts with 200 μg/ml GTE in the presence or absence of 1 mM H_2_O_2_ and a non-toxic dose (25 μM) of ZnPP9. GTE stimulation leads to a significant 1.5-times increase of HO-1 protein synthesis in the osteoblasts compared to the unstimulated control (Figure 6A). H_2_O_2_ stimulation reversed this effect, leading to a 1.4-time lower HO-1 protein synthesis compared with the GTE stimulated cells. The pre-incubation with GTE could slightly rescue the HO-1 protein synthesis. Additional it became evident that the presence of ZnPP9 significantly diminished the protective effect of GTE.

## Discussion

In the present study, we analysed whether the application of GTE can reduce or even prevent the negative effects on primary human osteoblasts caused by oxidative stress as occurring during inflammation. It was already reported that oxidative stress accelerates osteoclastogenesis and bone resorption, especially in elderly people [11,33,34]. Imbalances in the redox metabolism and altered mitochondrial oxygen utilization have been implicated in the overproduction of ROS. It plays a crucial role in the pathogenesis and etiology of several diseases, such as cancer, chronic inflammation or osteoporosis [35-37]. In the present study, we selected hydrogen peroxide as direct inducer of oxidative stress in primary human osteoblasts. This is a potent ROS activator as has been reported before [8,11].

We demonstrated that the GTE used for the experiments could be toxic for primary human osteoblasts in high concentrations after 24 h stimulation. However, all beneficial effects of GTE on prevention of oxidative stress in osteoblasts were observed in short-term experiments up to 4 h where GTE shows no toxic effects on cells. This has also been described by Park et al. [38], who showed that stimulation of cultured rat calvarial osteoblasts with 200 μg/ml green tea polyphenols did not affect the cell growth and viability for a short period. As reported before, green tea and its components are well-tolerable and rapidly absorbed by blood after oral administration in humans. Even after an intake of single high doses like 1.600 mg of epigallocatechingallate, the elimination in the blood plasma occurs after 5 - 6 hours post administration [39,40]. The short half-life of active GTE compounds also guarantees no accumulation risks after multiple administrations [41]. As shown in the present study, repeated stimulation of primary human osteoblasts with low doses of GTE over 21 days has positive effects on the cell function.

Oxidative stress is frequently associated with chronic inflammation. Therefore, it is of high interest, whether administration of green tea is beneficial when given prophylactic, simultaneously or therapeutic in combination with increased ROS levels. Therefore, we chose three different treatment possibilities to test the antioxidant capacity of GTE in primary human osteoblasts. The pre-incubation setting is simulating a prophylactic application of GTE, the co-incubation setting mimics acute situations and green tea post-incubation after induction of oxidative stress imitates the therapeutic approach. In all three settings, stimulation with high doses of GTE was able to protect the osteoblasts against oxidative stress. These protective abilities are probably due to the phenolic constituents of GTE. This is also reported by Chan et al., who show that the components of green tea significantly increase the free radical scavengers [19]. Shen et al. could already show a positive effect of green tea polyphenols resulting in improved bone volume, cortical thickness and bone mineral density in two rat models both suffering from osteoporosis (one female postmenopausal and one male) [25,42].

Improvements of bone strength and quality depend amongst other factors on bone mineralization. Therefore, we analysed whether repeated GTE application has any influence on the osteogenic differentiation process in vitro. Mineral matrix deposition increased constantly during 21 days of osteogenic differentiation. This is also reported by Vali et al., who show the positive effect of EGCG on the number and area of mineralized bone nodules in SaOS-2 human osteoblast [43]. In our study this process was delaying after chronic exposure to H_2_O_2_, which had been also reported by Arai et al. [8]. In contrast GTE in low concentration was able to improve the matrix formation, more valuable GTE complementation show protective effects against long term oxidative stress. Our findings are in line with results from Shen et al. [4], who show that chronic inflammation-induced bone loss in rats is caused by oxidative stress-induced damage and inflammation. In this study bone loss, measured by femoral mineral content and density, was stopped after dietary supplementation with green tee polyphenols. Additionally, other studies reported that green tea phenols such as EGCG are able to inhibit the expression of matrix metalloproteinase 9 (MMP-9) in murine osteoblasts, which prevent the degradation of organic and non-organic constituents of bone extracellular matrix [44]. The inhibition of MMP-9 expression in osteoblasts simultaneously inhibits osteoclast formation and additionally strengthened the bone structure [45-47]. We could also show that the expression of pro-osteoinducing genes such as osteocalcin and collagen1α1 by human osteoblasts during GTE application were improved. It was shown, that osteocalcin mRNA and synthesis correlates with calcium deposition in rat osteoblast [48]. Moreover, osteocalcin and collagen 1 promotes osteoblasts differentiation [49,50]. During the oxidative stress the GTE application of 1 μg/ml was able to recover the gene expression. As these genes are highly mandatory for bone building and bone strength [51,52]; GTE seems to be an important support for cell regeneration and defence against oxidative stress and bone catabolizing processes. We propose that the protective effect against oxidative stress of GTE is due to an increased expression of the anti-oxidative enzyme HO-1, as the addition of the HO-1 inhibitor ZnPP9 effectively blocked the protective effects of GTE on osteoblasts. Our results suggest that increasing HO-1 activity in osteoblasts protects them from ROI-dependent damage.

With this study evaluated findings contribute to our hypothesis that GTE improves bone formation and prevents bone loss. Similar effects were observed by Delaisse et al. [53]. They could show that, (+)-catechin an important antioxidative component of green tea could increase the resistance of collagen to collagenases in mouse calvaria explants. It prevents collagen degradation and bone resorption through osteoclasts. The possibility to halt and reverse the oxidative cell damage opens up new therapeutic opportunities. Patients, with increased oxidative stress levels suffering from chronic diseases, osteoporosis and delayed fracture healing could benefit from a GTE supplementation.

## Conclusions

We show positive effects of GTE on human osteoblasts in terms of scavenging of free radicals and decrease of inflammation. Simultaneously, GTE increases ECM production which might improve the bone quality. Therefore application of GTE might be a possible new therapeutic approach for treatment during inflammation-induced bone loss or prevention of osteoporosis and its complications by improving bone quality.

## Abbreviations

EC: Epicatechin; EGC: Epigallocatechin; EGCG: Epigallocatechin gallate; ECM: Extracellular matrix; GTE: Green Tea Extract; I/R: Ischemia/reperfusion; MMP: Matrix metalloproteinase; ROS: Reactive Oxygen Species; ZNPP9: Zinc protoporphyrine.

## Competing interests

The authors declare that they have no competing interests.

## Authors’ contributions

LS, AKN, CS and HV participated in the design of the study. LS, NH and CS performed the experiments. HV and MN collected the patient samples. LS, CS and AKN performed data analysis and interpretation of data and revised the manuscript. LS and CS prepared the figures. LS, CS and HV wrote the manuscript. All authors read and approved the final manuscript.
